# Multi-tissue metabolic and transcriptomic responses to a short-term heat stress in swine

**DOI:** 10.1186/s12864-024-09999-1

**Published:** 2024-01-23

**Authors:** Guilhem Huau, Laurence Liaubet, Jean-Luc Gourdine, Juliette Riquet, David Renaudeau

**Affiliations:** 1 GenPhySE, Université de Toulouse, INRAE, INPT, ENVT, 31326 Castanet Tolosan, France; 2 PEGASE, INRAE, Institut Agro, 35590 Saint-Gilles, France; 3 URZ, INRAE, Petit-Bourg, Guadeloupe France

**Keywords:** Heat stress, Pig, Multi-tissues, Multi-omics, Acclimation, NMR, Transcriptome, Metabolome

## Abstract

**Background:**

Heat stress (HS) is an increasing threat for pig production with a wide range of impacts. When submitted to high temperatures, pigs will use a variety of strategies to alleviate the effect of HS. While systemic adaptations are well known, tissue-specific changes remain poorly understood. In this study, thirty-two pigs were submitted to a 5-day HS at 32 °C.

**Results:**

Transcriptomic and metabolomic analyses were performed on several tissues. The results revealed differentially expressed genes and metabolites in different tissues. Specifically, 481, 1774, 71, 1572, 17, 164, and 169 genes were differentially expressed in muscle, adipose tissue, liver, blood, thyroid, pituitary, and adrenal glands, respectively. Regulatory glands (pituitary, thyroid, and adrenal) had a lower number of regulated genes, perhaps indicating an earlier sensitivity to HS. In addition, 7, 8, 2, and 8 metabolites were differentially produced in muscle, liver, plasma, and urine, respectively. The study also focused on the oxidative stress pathway in muscle and liver by performing a correlation analysis between genes and metabolites.

**Conclusions:**

This study has identified various adaptation mechanisms in swine that enable them to cope with heat stress (HS). These mechanisms include a global decrease in energetic metabolism, as well as changes in metabolic precursors that are linked with protein and lipid catabolism and anabolism. Notably, the adaptation mechanisms differ significantly between regulatory (pituitary, thyroid and adrenal glands) and effector tissues (muscle, adipose tissue, liver and blood). Our findings provide new insights into the comprehension of HS adaptation mechanisms in swine.

**Supplementary Information:**

The online version contains supplementary material available at 10.1186/s12864-024-09999-1.

## Background

Heat stress (HS) is one of the main climatic hazards that affect pig growth and reproductive performances [1], and welfare. In 2003, St-Pierre et al. [2] estimated elevated temperature results to annual losses near $300 million for the US swine industry, and this number rose to $900 million in 2018, according to da Fonseca de Oliveira et al. [3]. In temperate countries HS is an occasional challenge during summer heat waves. However, the frequency and intensity of heat waves are expected to rise in a context of climate change (IPCC 2023). HS is a constant problem in many tropical and subtropical areas where high ambient temperature effects can be accentuated by a high relative humidity. The part of pig production in tropical and subtropical areas has increased in the last 30 years and now represent more than half of the pig production, according to the United Nations for Food and Agriculture (FAO). When subjected to HS, pigs decrease their average daily feed intake (ADFI) to limit heat production [4]. Both the direct effects of HS and the associated effect of feed restriction induce various strong physiological and metabolic changes, the mechanisms of which remain poorly understood. The use of mRNA expression profiles and other omics data provides a powerful tool for identifying the molecular mechanisms underlying the physiological characteristics of heat adaptation in farm animals. Most published studies on the effects of HS on pigs based on omic traits focus only on one tissue, mostly lean muscle [5, 6], as it is of primary importance for the industry or the blood [7]. The hypothalamic-pituitary-adrenal axis is also well studied, as it is known to be important in the endocrine response to thermal stress. However, HS affect each pig tissue in different ways [8–10]. In fact, the animal’s response to HS is a complex and coordinated process that involves multiple subcellular compartments and multi-level regulatory pathways that are synchronized to avoid cell damage while maintaining cellular homeostasis [10–12]. Understanding the mechanisms behind HS adaptation is critical to address current and future issues in the industry. Apart from some reviews summarizing data from various studies, to our knowledge, there is no paper that provides an overview of the effect of HS on growing pigs through multiple omics and tissues in a single experiment.

The objective of this study was to have a better understanding of the difference and similarities of the adaptation mechanisms of pigs to HS in eight different tissues and fluids. We performed a multi-tissue analysis of the effect of a constant temperature of 32 °C during 5 days on growing pigs, with transcriptomic data from seven tissues (muscle, adipose tissue, liver, blood, thyroid, pituitary, and adrenal glands) and metabolomic data from four tissues (muscle, liver, plasma, and urine). We explored the effects on each tissue through differential expression and pathway enrichment analysis, and the relationships between tissues through correlation between mRNA and metabolite abundance.

## Results

Although animals of different breeds were included in the experimental design, this paper focuses only on the effect of ambient temperature on pig responses. Based on the statistical approaches used in the study, mean responses regarding the effect of the ambient temperature were corrected for the breed effect, and we checked for the absence of significant interactions between breed and temperature in our model.

### Animal responses to heat stress

**Fig. 1:**
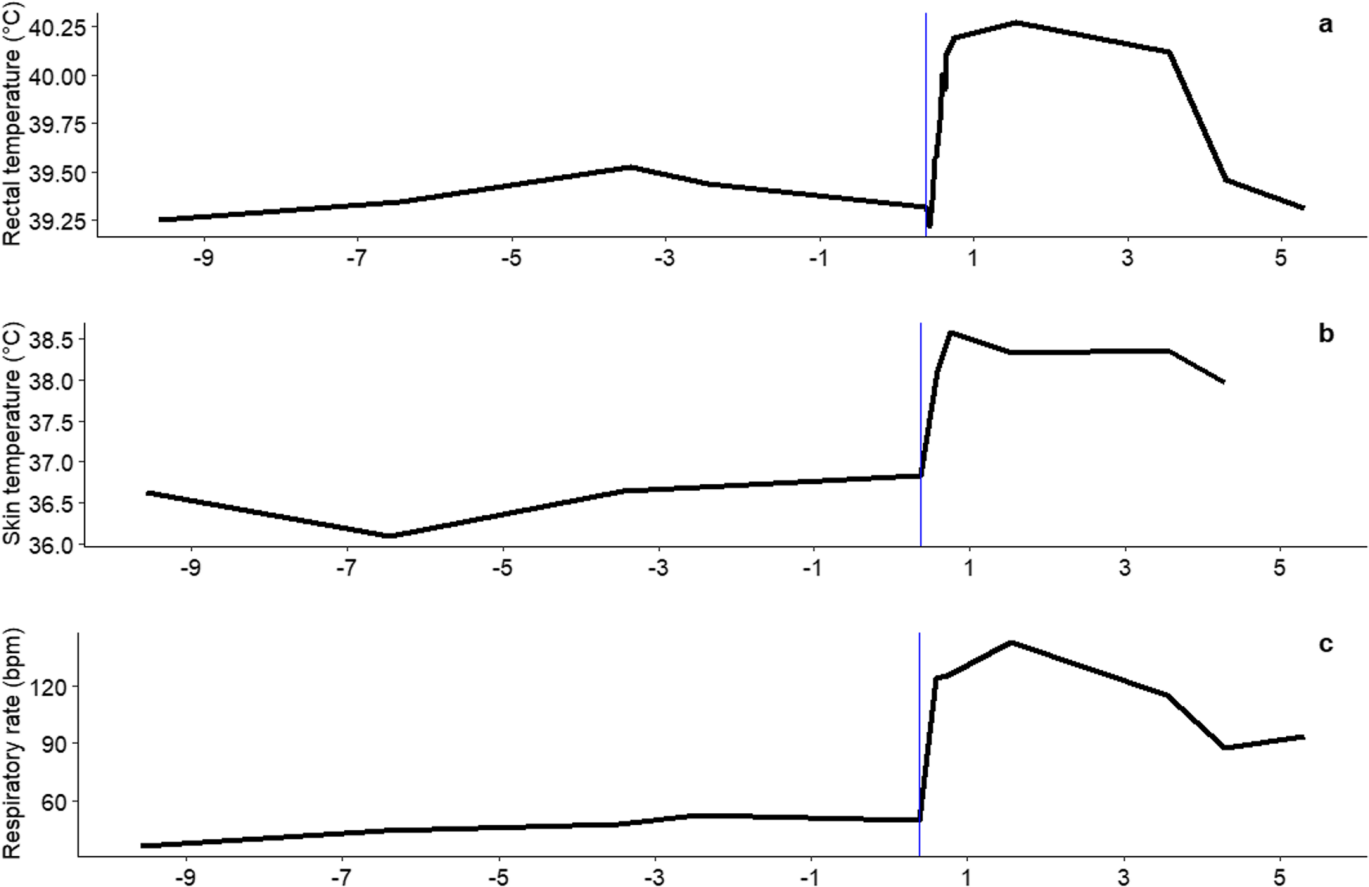
Rectal and cutaneous temperatures, respiratory during the experiment. The line is a mean of the variable for all pigs present at each period (*n* = [36-27-18-9]). The blue line represents the initiation of the change in ambient temperature from 24 °C to 32 °C **A** Rectal temperature. **B** Skin temperature. **C** Respiratory rate

The effects of elevated temperature (32 °C) on thermoregulatory responses are shown in the Fig. 1. The rectal and skin temperatures and respiratory rate significantly increased within the first 24 to 48 h of exposure to 32 °C when compared to the thermoneutral (TN) conditions (*P* < 0.01). This first increase was followed by a gradual decrease in these parameters indicating that acclimation responses have been implemented to reduce the HS relief experienced by the pigs. In addition, based on records of the 18 pigs that were subjected to the thermal challenge, the ADFI was significantly lower in HS at d3 conditions than in TN conditions at d-3 (1301 vs. 1829 g/d; *P* < 0.01).

As shown in Table 1, among the fifteen metabolites and hormones measured in blood plasma, only the triiodothyronine (T3), albumin and glycerol concentrations were significantly lower in HS conditions (*P* < 0.05). Tetraiodothyronine (T4), creatinine, and lactate concentrations displayed a tendency to be influenced by the climatic conditions (*P* < 0.10). Finally, although the effects were not significant, insulin and free fatty acids contents were numerically lower (− 22% and − 41%, respectively) in hot conditions.

**Table 1:** Mean and standard deviation (SD) of plasma metabolites and hormones concentration in TN (24 °C, *n* = 18 pigs) and HS conditions (32 °C, *n* = 18 pigs)

	Thermoneutrality		Heat Stress		Signif.
	Mean	Sd	Mean	Sd	
T4, μg/dL	3.73	0.42	3.45	0.42	0.07*
T3, ng/dL	52.4	13.1	44.3	13.6	0.05**
IGF-I, ng/mL	170	44.9	163	68.7	0.52
Insulin, μU/mL	7.4	5.18	5.78	2.6	0.63
Creatinine, mg/L	11.0	1.4	12.1	1.9	0.08*
Albumin, g/L	40.2	2.1	36.8	2.8	*<*0.01**
α-amino acids, mg/L	685	57	709	45	0.23
Proteins, g/L	64.8	4.8	62.1	6.7	0.18
Uric acid, mg/L	2.22	0.51	2.14	0.39	0.75
Urea, mg/L	290	83	283	65	0.89
Glycerol, mg/L	6.49	3.13	4.03	0.99	*<*0.01**
Triglycerides, mg/L	441	144	438	133	0.97
Free fatty Acids, μmole/L	267.3	265.9	160.9	86.1	0.15
Glucose, mg/L	1029	142	1000	71	0.30
Lactate, μmole/L	4718	2316	3396	1378	0.09*

* *P* < 0.1 ** *P* < 0.05

### Transcriptomic analyses of seven tissues: three regulators and four effectors

**Fig. 2:**
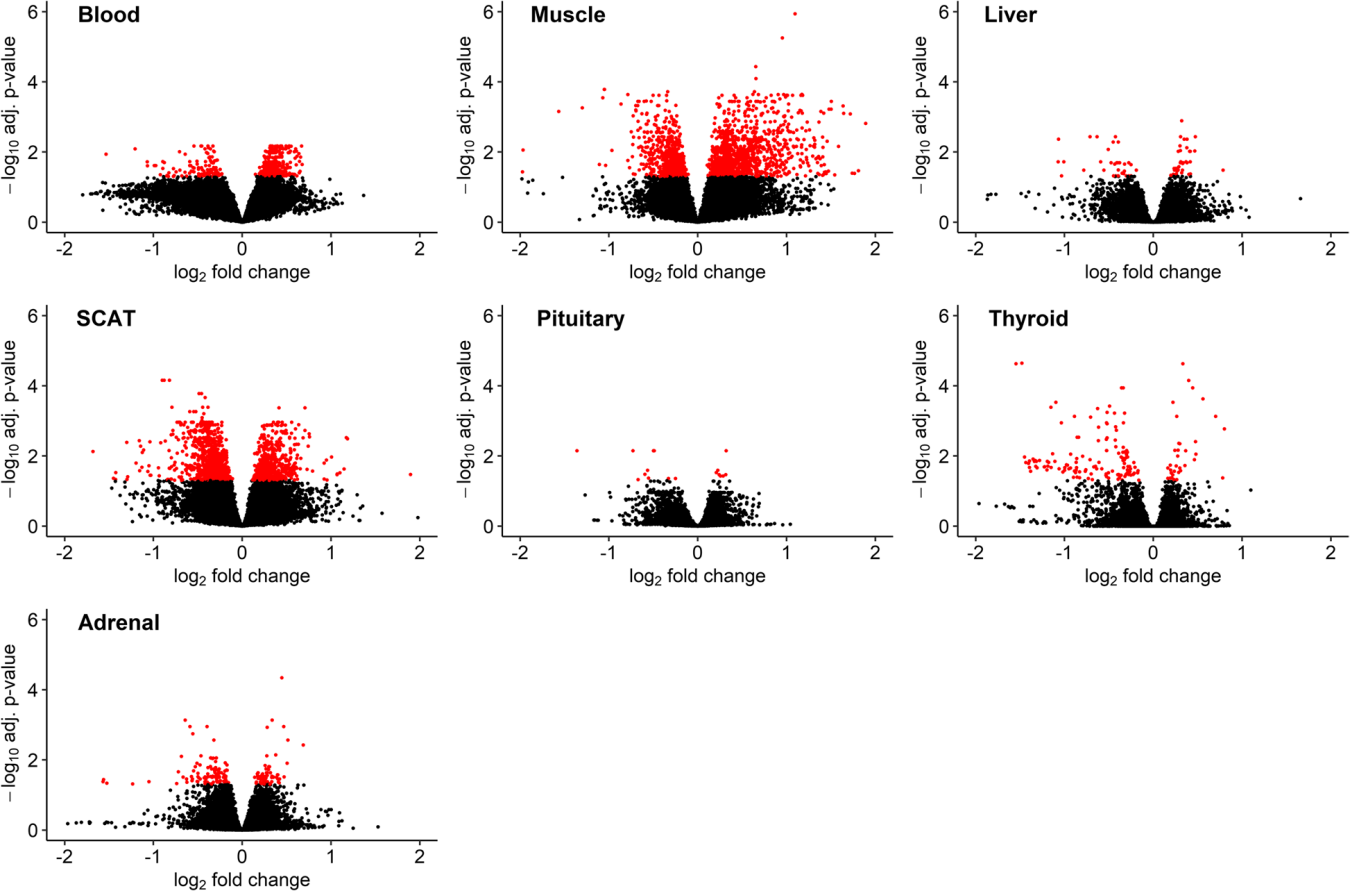
Volcano plots of the identified DEGs in response to heat stress. The gene expression level is represented on the x-axis through the log_2_ fold change. The y-axis is the *−* log_10_ adjusted *p*-value. Genes with an FDR adjusted *p*-value *<* 0*.*05 are shown in red. Results for the seven tissues are shown here: blood, liver, muscle, the subcutaneous adipose tissue (SCAT), the pituitary, the thyroid and the adrenal glands, respectively

An analysis was performed to compare the gene expression between HS and TN conditions for seven tissues (blood, liver, muscle, SubCutaneous Adipose Tissue (SCAT), pituitary, thyroid and adrenal glands) separately. A common preprocessing step was applied on each tissue independently. Genes with an FDR adjusted *p*-value lower than 0*.*05 were considered as differentially expressed genes (DEGs). A total of 481, 1774, 71, 1572, 17, 164 and 169 probes were detected as differentially expressed in blood, muscle, liver, SCAT, and in the pituitary, thyroid and adrenal glands, accounting respectively for 331, 920, 44, 741, 13, 82 and 88 unique annotated genes (Fig. 2). List of DEG for each tissue is available in Additional file 1. Expression of several important genes for HS is shown in Additional file 2.

**Fig. 3:**
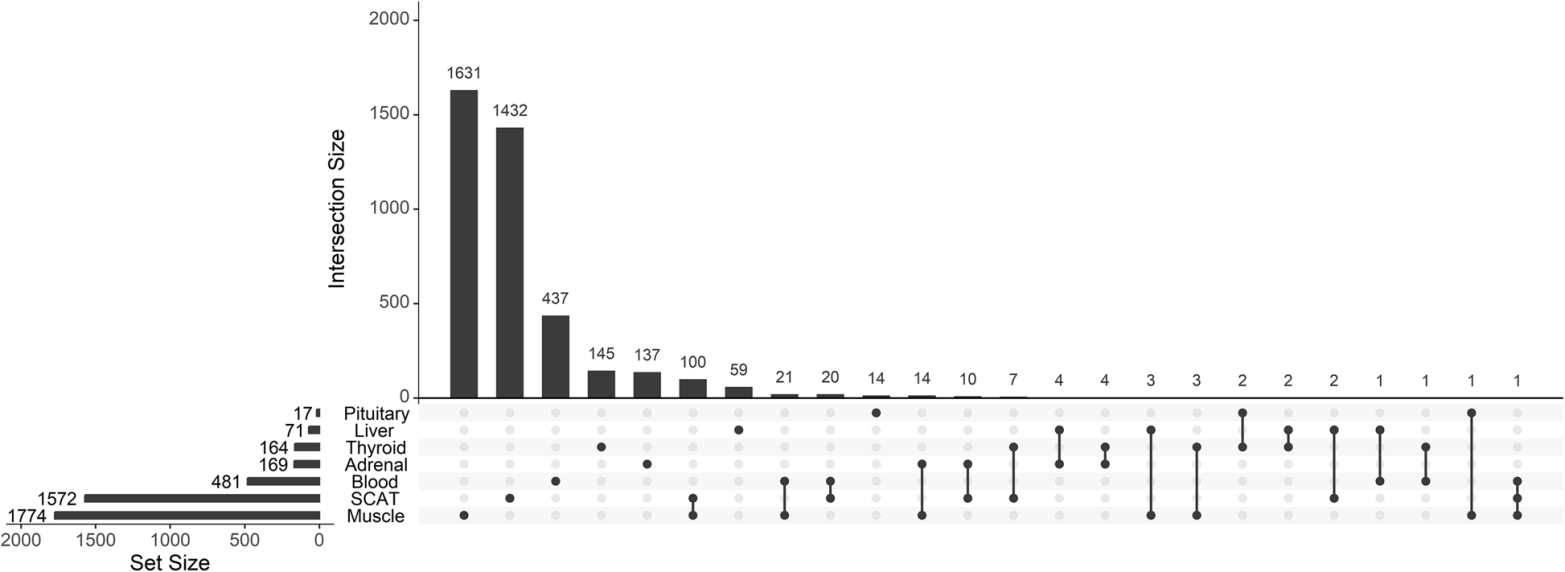
UpSet plot of the distribution of differentially expressed transcripts in the seven tissues

While 96.5% of genes expressed in one tissue are also expressed in another, 90% of DEGs are expressed in only one tissue accounting for 95% of the differentially expressed probes. Among the DEG of the muscle, we can cite *PDHA1, DLD, LDHB* and *HIF1A* involved in Glycolysis/Gluconeogenesis and/or HIF1 signaling pathway. Also genes involved in Thermogenesis or Reactive Oxygen Species such as *SOD1*, *HIF1A, ATP5ME* or *ATP5MF* both down or up regulated. In the SCAT, we found various down-regulated genes involved in fatty acid metabolism, e.g. *ELOVL6, FASN, LPL*. As shown in the Fig. 3, most of the shared regulated transcripts are common between the muscle and the SCAT (100 of 4053 probes), and to a smaller extent between the muscle and the SCAT with the blood (20 and 21 of 4051 probes respectively), suggesting a difference between these tissues and the others. Among the 10% of differentially expressed genes shared by multiple tissues, the HSP protein-coding family genes are well known to be involved in heat shock response. Specifically, *HSP10*, *HSP40*, and *HSP90* are differentially expressed in muscle, *HSP40* and *HSP70* in SCAT, *HSP40* and *HSP70* in blood, *HSP70* and *HSP110* in thyroid, *HSP70* and *HSP110* in adrenal glands, and *HSP110* in liver.

The enrichment analysis was performed on each individual tissue (Additional file 3). In blood, the main enriched functions are *carbon metabolism, glycolysis/gluconeogenesis, 2-Oxocarboxylic acid metabolism, histidine metabolism, phagosome* and *lysosome*. In the liver, enriched functions are all linked to fatty acid metabolism. No enriched pathways were found in the pituitary. The *p53 signaling pathway* and *pyrimidine metabolism* appear in the thyroid, along with other enrichments linked with the cell cycle. *Cortisol synthesis and secretion* and *p53 signaling pathway* were enriched in the adrenal gland. *Oxidative stress* together with *oxidative phosphorylation* and *reactive oxygen species* are enriched in muscle and SCAT in response to HS.

**Fig. 4:**
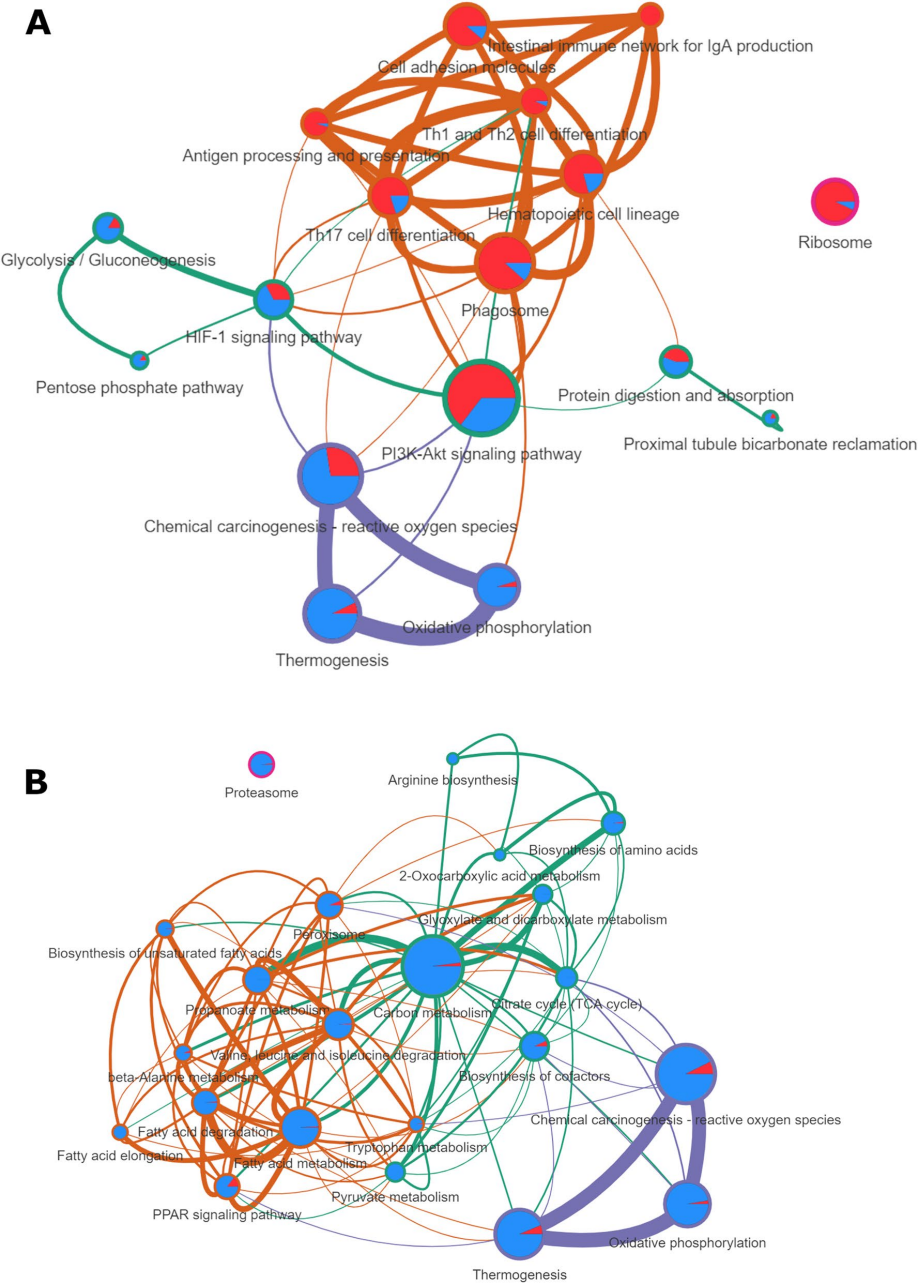
Enrichment network. **A** Enrichment network for the muscle. The orange cluster brings together terms related to the immune system. Purple cluster links *thermogenesis* and pathways related to oxidative stress. The green cluster is more related to energy homeostasis regulation. **B** Enrichment network for the SCAT. A purple cluster brings together *Thermogenesis* and pathways related to oxidative stress. A green cluster is mainly associated with energy metabolism pathways, and an orange cluster is more related to fatty and amino acid (AA) pathways. The size of the edges is proportional to the number of genes in common between two terms. The size of the nodes represent the number of DEGs in this term. A pie chart inside each node represents the proportion of up-regulated (red) and down regulated (blue) genes

A network of the enriched pathways for the muscle can be found in the Fig. 4a. Pathways can be clustered in three main groups: *immune response, thermogenesis/oxidative stress,* and *carbon metabolism/response to hypoxia*. A similar analysis was performed in the adipose tissue (Fig. 4b) in where three clusters of enriched pathways are shown in. They were related to the *thermogenesis/oxidative stress*, *fatty acid metabolism*, and *carbon metabolism*.

### Metabolome differential analysis

The ASICS R package [13–15] allowed to identify and perform a relative quantification of metabolites from 1H-NMR spectra in four tissues. A total of 104, 167, 83, and 110 metabolites were identified in plasma, urine, muscle, and in liver, respectively. Thanks to the measurement of some plasma metabolites (i.e., glucose, glycerol, lactate and creatinine) using “reference” methods, we were able to assess the reliability with quantification coming from ASICS. As shown in the Additional File 5, metabolites quantified by ASICS were correlated with those measured by reference methods. We obtained correlations of 0.98, 0.88, 0.79 and 0.65 for the lactate, creatinine, glycerol and glucose, respectively.

**Fig. 5:**
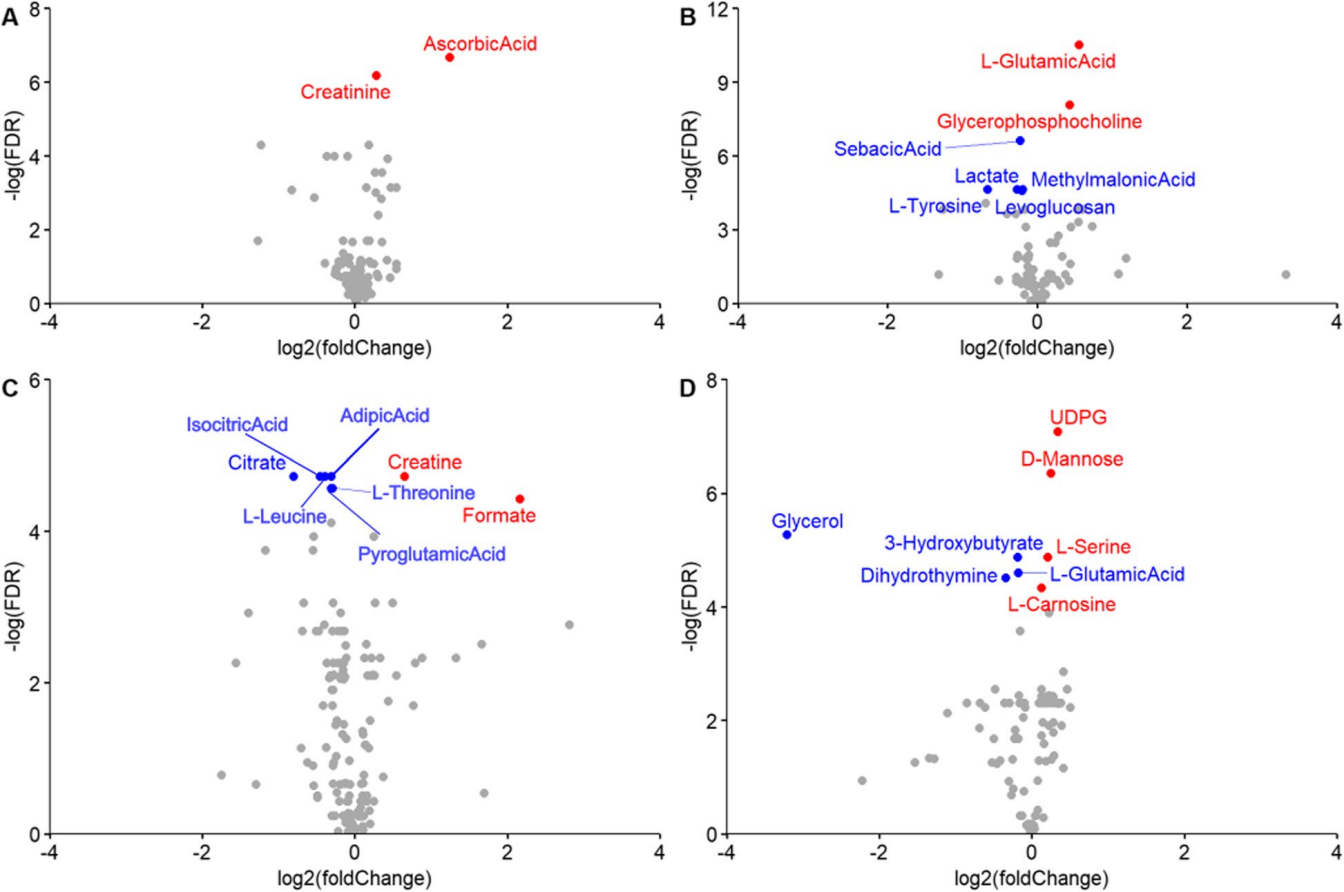
Volcano plot of the differential produced metabolites for **A** plasma, **B** muscle, **C** urine and **D** liver. Metabolites in red have an FDR adjusted *p*-value < 0.05 and are up regulated, and whose in blue are down regulated

After 5 days at 32 °C, in plasma, two metabolites were found to be significantly affected by the temperature, i.e., creatinine and ascorbic acid (Fig. 5a). In the muscle (Fig. 5b), seven metabolites (L-glutamic acid, glycerophosphocholine, sebacic acid, lactate, L-tyrosine, methylmalonic acid and levoglucosan) were differentially produced. In both urine and liver (Fig. 5c and d), we detected eight metabolites differentially produced. Isocitric acid, adipic acid, L-leucine, citrate, creatine, L-threonine, pyroglutamic acid and formate in urine samples, and UDP-glucose, D-mannose, glycerol, 3-hydroxybutyrate, L-serine, L-glutamic acid, dihydrothymine and L-carnosine for liver tissues.

### Focus on oxidative stress and thermogenesis through multi-omics analysis

**Fig. 6:**
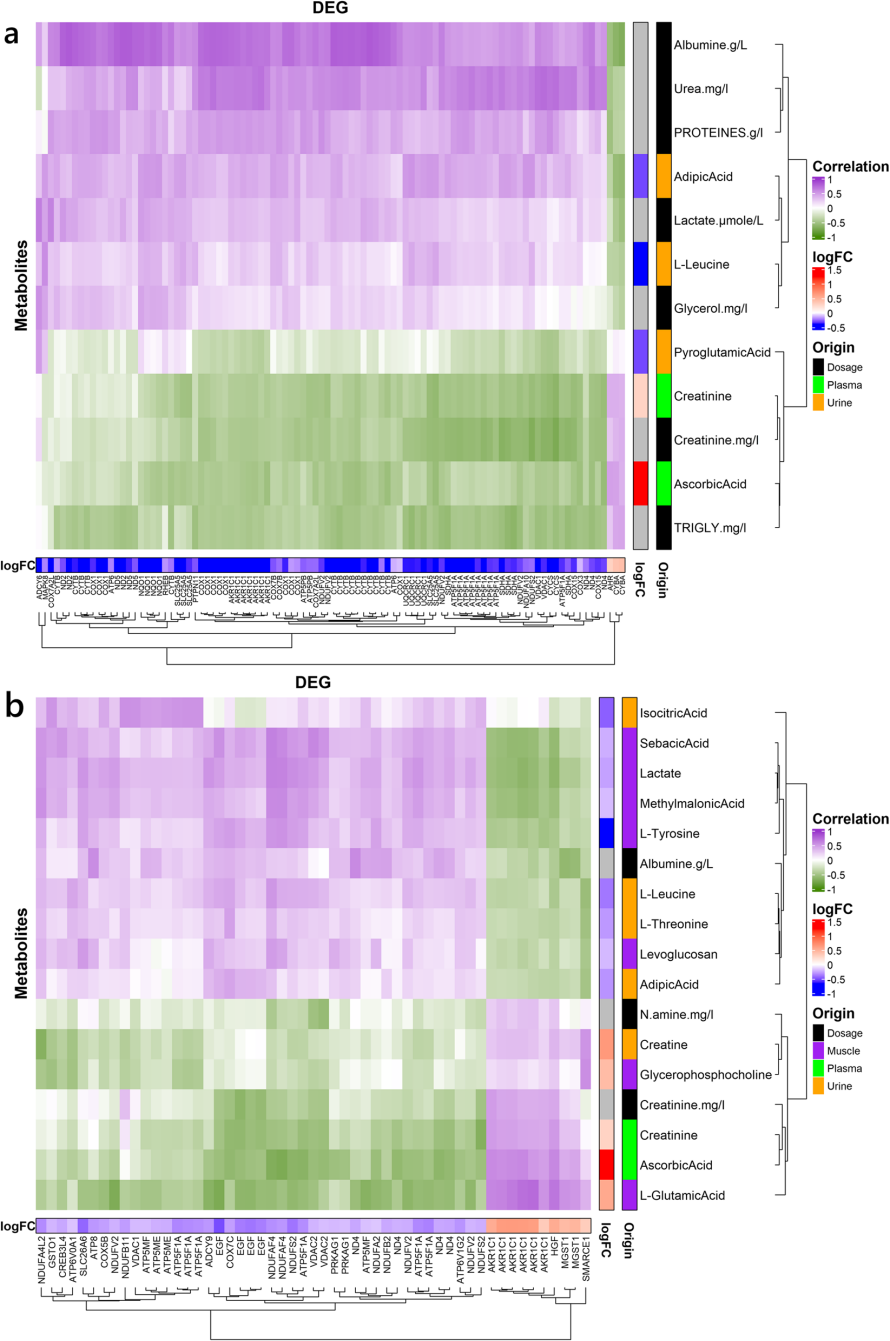
Heatmap of the correlation between genes involved in the oxidative stress and metabolites for the muscle (**a**) and the SCAT (**b**). The rightmost side annotation gives the origin of the metabolite quantification, while the bottom and left most side annotations give the log fold change of the transcripts and the metabolites respectively. We only show the transcripts and metabolites with at least one absolute correlation > 0.5. Duplicate names of a same gene correspond to different probes from the micro-array

As oxidative stress is often associated with HS, a focus was made on the genes involved in three pathways (i.e., *oxidative phosphorylation, thermogenesis* and *chemical carcinogenesis-reactive oxygen species*) both in *longissimus dorsi* (LD) muscle and SCAT tissues. A heatmap (Fig. 6) illustrating the correlation (> 0.5) between the DEG involved in these three pathways and the differentially produced metabolites and hormones from blood dosages and from the metabolome data measured with ASICS automatic quantification. These correlations are shown in Fig. 6a and b, respectively for (LD) muscle and SCAT. The heatmap for the muscle shows metabolites and genes clustered in two groups. In LD muscle (Fig. 6a), about half of the probes of the first cluster correspond to *aldo-keto reductase family 1* (*AKRF1*) transcripts, possibly involved in oxidative stress response. This group is also composed of *hepatocyte growth factor (HGF)*, *microsomal glutathione S-transferase 1 (MGST1)* and *BRG1-Associated Factor 57 (SMARCE1)*. This cluster of genes is positively correlated with protein catabolism biomarkers such as plasmatic N-amine and creatinine. The other cluster brought together genes involved in the mitochondrial respiratory chain (e.g., *NADH ubiquinone oxidoreductase, cytochrome c oxidase, ATP synthase*) and cAMP signaling. This second cluster is positively correlated mainly with muscle lactate content. Surprisingly methylmalonic acid, often associated with protein breakdown is negatively correlated with the first cluster and positively with the second cluster. In Fig. 6b, the heatmap for SCAT displays a main group of genes positively correlated with e.g., plasmatic lactate and albumin, and negatively correlated with e.g., plasmatic triglycerides. This cluster included genes also related to pathways including oxidative phosphorylation and apoptosis. A smaller group of genes can be observed with opposite correlations to the above-mentioned metabolites, including *aryl hydrocarbon receptor (AHR)* and *cytochrome b-245 α-chain (CYBA)* transcripts. One exception is the gene *MAPK6* which has a negative correlation with some metabolites related with proteins, e.g., plasmatic creatinine and proteins, and is positively correlated with the others.

## Discussion

Due to their limited ability to sweat, pigs are among farm animals the most susceptible to HS [16, 17]. In this study, based on combined measurements on the respiratory rate, skin and rectal temperatures, the exposure to high ambient temperature induced a bimodal thermoregulatory response, with an acute response in the first 2 days followed by a second phase of acclimation. These results agree with previous studies in swine [18] and in ruminants [19]. In addition, pigs subjected to 32 °C experienced a significant reduction in their voluntary feed intake. This drop in feed intake during the 5-d exposure period to 32 °C (− 18.4 g.d^−1^.°C^−1^) is rather similar to the value previously reported in pigs within the same temperature range by Renaudeau et al. [4]. As reported in this later study and by many other authors, reduced feed intake in hot conditions is the most effective way to lower the heat load for pigs and allows maintaining their core body temperature within physiological limits. Overall, our results indicate that blood, urine and tissue samples collected after 4 or 5 days of exposure to 32 °C were obtained in pigs that already initiated heat acclimation responses.

As suggested by Collier et al. [20], heat acclimation is a homeorhetic that involves different tissues. This study is a first attempt in pigs to assess how regulatory and effector organs interact together or independently with the aim of achieving a heat acclimation state. Depending on the effector tissues (muscle, SCAT, liver, and blood) the number of DEGs in a five-day HS condition varied widely. The LD muscle has the highest number of DEG whereas liver exhibited the lowest number of DEG. These results are consistent with those of Lagarrigue et al. in poultry [personal communication]. They suggest that muscle tissue may have a unique and lasting sensitivity to HS. Alternatively, muscle could be the key effector tissue of the acclimation responses to HS. As quantitatively the largest organ in the body, skeletal muscle account for 20% of the total heat production in fasting growing pigs [21] and have a significant influence in the whole body energy metabolism and/or fluxes of energetic substrates. The skeletal muscle exhibits remarkable metabolic flexibility in fuel usage in response to various metabolic challenges such as energy deprivation and changes in diet composition. HS conditions can negatively impact muscle metabolism due to reduced energy supply and direct heat effect. Studies show that key enzymes involved in metabolic pathways such as glycolysis (6 genes), TCA cycle (6 genes), and respiratory chain (16 genes, mainly for Complex 1) are reduced under such circumstances, including the pyruvate dehydrogenase (PDH) complex. The PDH complex plays a crucial role in regulating metabolic flexibility by selecting glucose, fatty acids, and proteins for cellular energy production. *PDK4* expression is up-regulated in rats [5] and pigs [6, 22] under high-temperature conditions, suggesting a shift towards fatty acid oxidation and/or glycolysis. However, our study shows that in such conditions, the *PDK4* gene expression is down-regulated in muscle, suggesting that carbohydrate use is favored over lipid oxidation.

Under an acute stress response phase and/or a very intense short-term effort, oxidative phosphorylation is the major ATP generating pathway and glucose liberated from glycogen is generally a predominant fuel source. In the present study, the down-regulation of both *PYGM* (Glycogen Phosphorylase, Muscle Associated) and *PHKA1* (a phosphorylase kinase) genes, suggests a reduction in the use of intramuscular glycogen after 4 to 5 days of exposure to 32 °C. Similar results were reported in pigs after a 21-d exposure period to 30 °C [6]. In addition, we also found a reduction of the expression of the glycogen synthase (*GYS1*) and an accumulation of an important substrate for glycogenogenesis (i.e., UDP-glucose) in the liver in HS conditions suggesting that glycogenesis is prevented after 5 days of HS. In fact, during a prolonged feed restriction period, glycogen is depleted, and glucose is produced from available non-carbohydrate carbon substrates as precursors of gluconeogenesis (amino acids, lactate, and glycerol) mainly in the liver but also in the kidney and intestine [23].

In the present study, glycerol hepatic and blood levels were significantly lower in HS than in TN conditions. In addition, the hepatic glycerol-3-phosphate dehydrogenase (*GDP1*) gene was down regulated under HS. Due to its implication on the pathway of using glycerol as a substrate of gluconeogenesis, that implies that pigs do not rely on glycerol to produce glucose under HS condition. This result could be related to the reduced availability of blood glycerol in connection to lack of adipose tissue lipolysis in HS conditions. In rodents, the use of glycerol as a neoglucogenic substrate has been shown to be significant only at the beginning of the heat exposure period [24]. In other words, we can hypothesize that the very low hepatic glycerol content measured after 4 to 5 days at 32 °C was a consequence of an extensive use of glycerol as a substrate for gluconeogenesis in the first hours or days of heat exposure. Further studies are needed to verify this assumption.

In agreement with the results of Lebret et al. [25], HS pigs showed a tendency to have a lower lactate blood concentration. As lactatemia results from an equilibrium between lactate production and consumption, we can first suggest that hypolactatemia measured in the present study may result from the hepatic regeneration of glucose from lactic acid produced in muscle as a product of anaerobic metabolism, via the Cori cycle. However, our results indicated a reduced lactate concentration and an up-regulation of the lactate dehydrogenase A (*LDHB)* gene in muscle of HS pigs. In contrast with our findings, Yang et al. [26] reported an increase in muscle lactate content after a 3 week of exposure to 30 °C. In addition, Sanders et al. [5] showed that *LDHA* gene expression tended to increase in the soleus muscle after a 6 h-period exposure to 39.4 °C in rodents, whereas Hao et al. [6] reported a significant down regulation of *LDHA* gene expression in pigs exposed to a constant HS (30 °C) for 21 days. Finally, Sanz Fernandez et al. [8] failed to show a significant *LDHA* gene expression change when pigs fed ad libitum and kept at 32 °C for 8 days were compared with pair-fed pigs kept at thermoneutrality. The discrepancy between these different results may be related to variations in lactate accumulation in postmortem muscle in connection with differences in the duration of fast prior to the slaughter or to the duration of HS exposure. We can also assume that changes in muscle lactate concentration and *LDH* gene expression observed in the present study would be related to the reduced energy intake rather than a direct effect of elevated temperature. When compared to the *LDHA*, *LDHB* enzyme performs a bidirectional of the conversion of pyruvate and lactate with an higher affinity for lactate than pyruvate [27]. This could suggest that lactate is used directly as an energy substrate in HS conditions. Lebret et al. [25] suggests that an increased activity of *LDH* could be related to an adaptation to acute HS, whereas a reduced muscle glycolysis occurs during long-term exposure. A longitudinal study and a per-fed TN group could help to unravel the origin of these mechanisms and determine if lactatemia is more influenced by feed restriction or HS, while accessing to a better understanding of its temporal pattern.

As the largest reservoirs of amino acids (AA), muscle protein metabolism directly affects the whole-body nitrogen metabolism. In the present study, the expression of the gene *BCAT1* (Branched Chain Amino Acid Transaminase 1) in LD muscle (i.e., an enzyme considered as the first step for the catabolism of branched-chain AA) was up-regulated, and the glutamic acid muscle content increased in HS conditions. In addition, biomarkers of muscle protein turnover (i.e., plasmatic and urinary creatinine [28]), tended to increase in HS pigs in our study. As demonstrated in poultry [29], HS caused muscle protein breakdown to provide AA substrates to liver gluconeogenesis responsible for energy supply. In the present study, the muscle *MLST8* gene coding for a subunit of mTORC complexes (involved in the regulation of protein synthesis) was found down regulated at 32 °C in agreement with a review of Ríus on cattle [30]. Growth of muscle mass is achieved by a daily net protein balance between muscle protein synthesis rates and muscle protein breakdown rates. From that, it can be hypothesized that in our experimental conditions, HS resulted in reduced muscle growth. This conclusion agreed with that of Le Bellego et al. [31], which indicated that maximal protein deposition is considered to be limited in hot conditions.

The adipose tissue, responsible for storing excess metabolic energy in the form of fat, had the second-highest number of DEGs in this study. The energy stored as fat can be then mobilized during periods of energy deprivation (hunger, fasting, and diseases). In hot conditions, direct and indirect consequences will have contrary effects on adipose tissue growth. Under an energy deficit status caused by feed restriction, fat deposition is generally reduced. In contrast, in connection with the direct effects of HS on protein deposition, fat accretion can increase in particular feeding conditions [32]. In our study, most DEG involved in both lipid anabolism and catabolism in the SCAT were down expressed in HS conditions. In particular, genes associated with lipid anabolism (such as fatty acid synthase *FASN* or fatty acid elongase *ELOVL6*) are down regulated in backfat samples of HS pigs. However, as back fat is not the only site for lipid storage in the carcass, our results do not allow us to conclude on the absence of a lipid deposition in HS conditions. In fact, some previous studies have shown as increased fat accretion in internal perirenal tissues at the expense of backfat [32–34]. According to our results, lipoprotein lipase (*LPL*) gene was upregulated in agreement with Kouba et al. [32] and other studies [35, 36] suggesting that SCAT adipose tissue would be a potential energy supplier in HS conditions. Generally, in pigs facing a dietary restriction challenge, adipose tissue is mobilized and NEFA are used as an energy source [22]. In the present study, the plasma concentration of NEFA was numerically lower for HS pigs compared to TN despite a significant feed restriction. Additionally, their plasma levels of albumin (the main fatty acid transporter) and glycerol (a product of lipolysis) were significantly lower. These results suggest that adipose tissue may not be able to compensate for the energy deficit in these HS conditions. This conclusion agrees with those of previous studies [8, 33].

In both muscle and adipose tissue, our results revealed a strong enrichment of GO processes related to oxidative stress response, mitochondrial respiratory chain and other aspects of energy metabolism. Part of these responses are regulated by thyroid hormones, which are important (up)-regulators of the basal metabolism and also act on thermoregulation. Plasmatic levels of thyroid hormones were reduced by 8 and 15% for T3 and T4 in HS, respectively, in our study. A decrease in thyroid hormones (T3 and T4) is a known effect of HS [10, 33, 37] and is thought to be one of the reasons for the observed decrease in basal energy metabolism, as well as a decrease in lipolysis. Thyroxine-binding globulin (SERPINA7), the main transporter of thyroid hormones in the blood (bind 75% of serum T4), is mainly produced in the liver. In our study this gene was upregulated. Considering that this protein is not saturated in normal conditions [38] and that the levels of thyroid hormones decrease during HS, this result needs further investigation. However, this could be an adaptative mechanism to further reduce active (unbound) thyroid hormones level by binding more of them and reducing metabolic heat production by decreasing energy metabolism.

Another important regulator of the metabolism is insulin, a potent anabolic hormone that primarily regulates glucose uptake and stimulates lipogenesis and protein deposition. In addition, insulin plays a role in activating the HSP response [39]. In our experimental conditions, insulin level was not significantly different between TN and HS, possibly due to the high variations between individuals. While most studies have shown an increase in insulin levels during HS [33, 40], some have not found a direct difference in insulin levels but in insulin sensitivity [41]. It also seems that insulin plays a role in long-term adaptation to HS, as it has been reported in cattle that an increase in circulating insulin occurs after the first days of HS [42]. This could explain the lack of changes in insulin levels in our study.

Under our experimental conditions, regulatory tissues (i.e., pituitary, thyroid and adrenal glands) showed the lowest number of DEGs. From these results, one can be hypothesized that these tissues are less sensitive to the effects of HS than muscle, back fat or liver. However, as these tissues are involved in the immediate responses to HS, we cannot totally exclude that the main gene expression changes occurred before the 4th or the 5th days of acclimation. At the transcriptomic level and at the time of sampling, the pituitary-adrenal and pituitary-thyroid axes are the least affected by HS among all our tissues. Adrenal glands are also involved in reaction to various stressors. For example, in thyroid, DEG associated with pyrimidine metabolism i.e., cell death and apoptosis, were down regulated. Interestingly in adrenal glands, DEG related to environmental stress [Growth Arrest and DNA Damage Inducible Alpha (*GADD45A*), heat shock protein 70 (*HSPA8*), heat shock protein 110 (*HSPH1*) and Stress Induced Phosphoprotein (*STIP1*)] are down regulated. It is possible that due to their localization, function and importance, these three tissues are more protected from HS or affected earlier in response to HS compared to other tissues such as muscle or adipose tissue.

During exposure to HS, a highly conserved family of proteins named heat shock proteins (HSP) are produced. Among the HSPs, the two most expressed families of these chaperone proteins are *HSP70* and *HSP90*, which allow, alone or in association with *HSP40,* to stabilize and facilitate the proper folding of proteins under HS conditions. In our experimental conditions, *HSP70* was surprisingly downregulated in adipose tissue, thyroid and adrenal glands whereas it was upregulated in the blood. In the other hand, *HSP90* was up-regulated only in the muscle tissue whereas *HSP40* was down-regulated in muscle and adipose tissues but up-regulated in blood.. These results seem to indicate that each HSP as a tissue-dependent expression in accordance with previous findings [43–47]. As in the literature, different patterns of gene expression were found between HSP70 and HSP90 families in HS conditions, dependent of the tissue and/or the duration and level of HS [45, 48, 49], possibly being a proxy of a tissue-specific susceptibility to HS. In other words, we cannot totally exclude that tissue variations in the HSP expression on d4 and d5 could also reflect differences in their expression kinetics in the first days of exposure to 32 °C.

Excessive heat load leads to oxidative stress in poultry [50] and swine [51]. Oxidative stress is defined as an imbalance between the production of reactive oxygen species (ROS) and the antioxidant capacity of the cell. The accumulation of ROS causes damage to macromolecules such as proteins, lipids and DNA and alters their structure and function. The main source of ROS production within most cells is the mitochondria. Respiratory chain in the mitochondria enables the production of ATP by creating an electrochemical gradient across the inner membrane of the mitochondria, using different protein complexes. Higher temperatures were associated with a decrease in the expression of the respiratory chain genes, mostly for the Complex 1 in our study. Interestingly, in Complex 1, electrons are transferred from NADH (produced in the TCA cycle) to ubiquinone, and it is one of the primary sites where electron leaks can occur [52], resulting in the generation of ROS. The down expression of related genes in this study could be a mechanism to prevent ROS noxious effects during HS, in complement with the action of ROS scavengers. Both *reactive oxygen species* and *oxidative phosphorylation* pathways were enriched in the muscle, with in particular a slightly higher expression of the superoxide dismutase (*SOD*) in accordance with Montilla et al. [51]. According to these latter authors, the susceptibility to HS mediated changes in redox balance is lower in glycolytic than in oxidative muscle and the resolution of oxidative damages are very rapid after the onset of the thermal challenge (within the first 72 h). In fact, SOD prevents the accumulation of ROS by converting superoxide to hydrogen peroxide, which is then removed by glutathione peroxidase (downregulated in our study) or catalase. However, it seems that results in literature show variable results regarding these genes expression [53, 54]. Hypoxia-inducible factor (HIF) is a key regulator of the cellular response to oxidative stress during early HS exposure and was not activated in the muscle as suggested by a small down regulation of *HIF1α*. Moreover, peroxiredoxin (*PRDX4* in muscle and *PRDX3* in SCAT), a family of protein with antioxidant and chaperone properties, was found down regulated, similarly to another study [55], but upregulated in the blood (*PRDX1*). Contrary to the muscle, the fact that SCAT is not under oxidative stress in this study, could explain the down regulation of genes involved in oxidative stress such as *SOD* or *PRDX3*. Furthermore, of the two phospholipases that appeared to be differentially expressed, the one coding the phospholipase A2G7, which is known to degrade phospholipids damaged by oxidative stress [56], is down regulated. Like the SCAT, liver does not seem to be subject to oxidative stress. A reason could be the presence of a higher quantity of carnosine, a dipeptide with antioxidant capacities [26, 57]. These results suggest that in our experimental conditions part of the antioxidant responses took place prior to the slaughter of our animals, i.e., before 4 or 5 days of HS. This is supported by the fact that acute HS studies have seen an increase in peroxiredoxin expression in the first hours of HS in pigs and poultry [58, 59]. Moreover, in contrast with the founding of Blincoe et al. [60] and Tanaka et al. [61] in cows, we found that high temperatures are correlated with a higher level of plasmatic ascorbic acid. Ascorbic acid has many functions and it can be difficult to assess the reason for its variations. Nethertheless, among its functions, the ones that could be interesting in HS conditions are antioxidant properties, carnitine biosynthesis, biosynthesis of corticosteroid hormones and use in the immune system [62]. Ascorbic acid has been successfully used as supplementation diet in multiple experiments to attenuate HS effects on broilers [63] and has also been found at decreased levels in lactating heat stressed cows [64], and could be present as a protection from oxidative stress in the plasma.

## Conclusion

In this study, the aim was to get a better understanding of the differences and similarities of the adaptation mechanisms of pigs to HS. Our experiment allowed us to compare gene expression and metabolite production for seven and four tissues respectively, between TN and HS pigs. We have identified different patterns of DEG between regulatory and effector tissues. Through a correlation analysis, we were able to unravel some possible interactions between tissues connected to oxidative stress during an HS challenge. The results of this study can help to discover novel or to better understand known mechanisms of adaptation to heat stress, as well as to select new biomarkers that could improve the selection of pigs with a higher resilience to high temperatures and that can be used for precision farming.

## Methods

### Animals and treatments

A total of 36 castrated male growing pigs (12 Large White, 12 Créole and 12 Large White×Créole) used in the present experiment, were raised in experimental facilities of the Tropical Platform for Animal Experimentation (PTEA) of the French National Institute for Agriculture, Food and the Environment (INRAE), in Guadeloupe. At 11 weeks of age, pigs were moved to a climatic room equipped with 12 similar slatted floor pens (1.5 × 2 m). We arranged the animals in blocks of 4 littermate pigs (3 blocks/breed). Pigs of the same breed were housed in groups of 3 (each from a different block) in the same pen. Then, there were 4 pens per breed. For each breed, 2 pens were slaughtered at 24 °C and 2 at 32 °C. Pigs were adapted to the experimental conditions (housing and diet) for 10 days. During this adaptation period, the ambient temperature was set at 24 °C. The experimental period was divided in two phases: pigs were kept at 24 °C for 9 days (phase 1, thermoneutral conditions, TN) and thereafter at a constant temperature of 32 °C for 5 days (phase 2, heat stress conditions, HS). Between these two phases, the ambient temperature was changed on d 0 from 24 °C to 32 °C at a constant rate of 2 °C/h beginning at 0900. The relative humidity (RH) was kept constant at 80%. The severity of heat stress (i.e., 32 °C) and the duration of exposure (i.e., 4–5 days) were determined on the basis of previous works [65] to induce physiological responses related to heat acclimation in all animals.

Pigs had free access to a commercial diet based on corn, soya-bean meal, and wheat middlings. Each pen was equipped with a feed dispenser and a nipple drinker designed to avoid water spillage. The photoperiod was fixed to 12:30 h of artificial light (from 0600 to 1830) and the ventilation rate was set at 50 m^3^·h^−1^·pig^−1^. Airspeed was not controlled, but periodical spot measurements at the animal level indicated that it did not exceed 0.15 m.s^−1^. Half of the pigs (*n* = 18, TN group) were slaughtered before the rise in ambient temperature on d-2 (*n* = 9) and d-1 (n = 9). Half of the pigs (n = 18, HS group) were slaughtered after four or 5 days of exposure to 32 °C. Additional Fig. 6 provides a diagram of the experimental design timeline.

### Phenotypic measurements

All pigs were weighed at d-11 and at slaughter. Feed intake was monitored daily in each pen. Respiratory rate (RR), rectal temperature (RT), and skin temperature (ST) were measured in all pigs at 1300 on days − 7, − 4, − 3, 0, + 1, + 3, + 4 of the experiment (Additional file 6). On d0, the same measurements were performed at 0900, 1400 and 1800 and RT was measured each half hour, from 1000 until 1400, and at 1500. These physiological measurements were performed according to the following protocol: first, RR was visually determined by counting flank movements over a period of 1 min, only in resting pigs. Then, RT was measured using a digital thermometer (Microlife Corp., Paris, France), and ST was measured on the back using a skin surface thermocouple probe (type K, model 88,002 K-IEC, Omega Engineering Inc., Stamford, CT, USA) connected to a microprocessor-based handheld thermometer (model HH-21, Omega Engineering Inc.). The RT and ST measurements were performed in unrestrained animals and with the minimum of stress. Pigs were slaughtered in four series of 9 pigs each without a previous fasting period. Pigs were euthanized with a slaughtering pistol (Matador, Termet, Champagné, France). Immediately after slaughter, 20 mL of blood was sampled during the exsanguination. Immediately after the sampling, 2.5 mL of blood was put into a PAXgene® RNA tube (PreAnalytiX, Qiagen). These tubes were inverted 10 times after collection and incubated at room temperature for 4 h for RNA stabilization, transferred to − 20 °C overnight, and stored at − 80 °C. The remaining blood was centrifuged at 2000 g and 4 °C for 10 min and plasma was stored at − 20 °C or − 80 °C depending of the analyses. Tissue samples were collected at the dorsal subcutaneous adipose tissue (back fat) location (last rib level) and from the *longissimus* muscle just below back fat sampling in the right carcass side. After opening the abdominal cavity, 40 mL of urine was collected directly in the bladder and tissue sections were immediately sampled from the left lobe of the liver, the anterior lobe of the pituitary, the whole thyroid and the whole adrenal glands. Tissue samples were immediately cut, frozen in liquid nitrogen, and stored at − 80 °C until analyses. Urine samples were homogenized and subsampled in Eppendorf tubes and stored at − 80 °C.

### Blood analyses

Commercially available kits were used to measure plasma levels of non-esterified fatty acids or NEFA (FUJIFILM Wako Chemicals Europe GmbH, Neuss, Germany), creatinine (Créatinine cinétique, Biomérieux, ref. 61,162), glucose (Glucose RTU, Biomérieux, ref. 61,269), and urea (Kit Urée cinétique, Biomérieux, ref. 61,974). Intra-assay CV were 3.71, 0.28, 4.25%, respectively. Inter-assay CV were1.75, 0.22, 2.66% respectively. Plasma levels of insulin (ST AIA-PACK IRI, Tosoh Corporation, Tokyo, Japan), total triiodothyronine or T3 (ST AIA-PACK TT3, Tosoh Corporation, Tokyo, Japan) and total thyroxine or T4 (ST AIA-PACK T4, Tosoh Corporation, Tokyo, Japan) were also determined. Intra-assay CV were 2.3, 3.8 and 3.9%, respectively. More detailed information on kits used in these analyses are given in Additional file 7.

### Proton nuclear magnetic resonance (^1^H-NMR) spectroscopy analysis

Generation and normalization of metabolomics spectra were carried out on MetaToul platform (Toulouse metabolomics and fluxomics facilities, www.metatoul.fr). Water-soluble metabolites were extracted from 100 mg tissue with 1 mL of ice-cold 80% (v/v) methanol and 0.6 mL acetonitrile. Plasma, urine, and water-soluble extract from muscle and liver samples (200 μL) were diluted with 500 μL of D2O and centrifuged at 5000 g for 10 min at 4 °C before they were placed in 5 mm 1H-NMR tubes. All 1H-NMR spectra were acquired on a Bruker DRX-600-Avance 1H-NMR spectrometer operating at 600.13 MHz for 1H resonance frequency using an inverse detection 5 mm 1H-13 C-15 N cryoprobe attached to a CryoPlatform (the preamplifier cooling unit). The 1H-NMR spectra were acquired at 300 K using the Carr-Purcell-Meiboom-Gill (CPMG) spin-echo pulse sequence with pre-saturation, with a total spin echo delay (2 nτ) of 240 ms to attenuate broad signals from proteins and lipoproteins. A total of 128 transients for liver, muscle, and plasma, and 256 transients for urine were collected in 32 K data points using a spectral width, a relaxation delay and an acquisition time of 20 ppm, 2.5 sec, and 2.28 sec, respectively. The spectra were Fourier transformed by multiplication of the free induction decay FIDs by an exponential weighting function corresponding to a line-broadening of 0.3 Hz. All spectra were manually phased and baseline corrected, and referenced to 3-trimethylsilylpropionate TMSP using the Bruker TopSpin 2.1 software (Bruker, GMBH, and Karlsruhe, Germany).

### Metabolite identification and quantification

All analyses were carried out using R version 4.2.1 (2022–06) [66]. 1H-NMR spectra were processed using the R package ASICS, version 2.14.0 [14]. Standard preprocessing of the spectra (as described in [14, 67]), including Free Induction Decay signals importation, water area removal, or baseline correction was applied. All the 144 spectra were then aligned using the joint alignment procedure described in [15]. Automatic quantification of metabolites were performed using the ASICS joint quantification procedure. Based on previous analyses on similar data [4], the noise.thres, add.noise, and mult.noise were set at 0.02, 0.15, and 0.172, respectively. In addition, the clean-up threshold (clean.thres) for the joint quantification procedure was set to 10%. This means that all metabolites identified in less than 10% of all samples were excluded during the quantification procedure. This choice was based on previous studies and validated on independent direct dosage of metabolites on the same samples, as described in [4]. Raw data, normalized data and metadata are available under https://entrepot.recherche.data.gouv.fr/dataverse/omics with the DOI accession 10.57745/HCFXBY.

### RNA isolation and microarray hybridization

Total RNA was extracted and quantified from individual samples with the Nucleospin RNA kit (Macherey Nagel) for all tissues (muscle, liver, adipose tissue, adrenal gland, pituitary, thyroid) except blood. Briefly, 60 mg to 100 mg of tissue was disrupted 2 min at 30 Hz in Trizol with an inox bead with the mixer Mill MM 400 (Retsch). After a 10 min centrifugation at 12,000 g and at 4 °C, RNA was isolated with chloroform, and precipitated with ethanol. An enzymatic treatment with DNAase was performed to avoid genomic contamination. RNA was eluted in 40 μL to 100 μL in RNAse-free water and stored at − 80 °C.

Total RNA from blood samples collected on PAXgene Blood RNA Tubes (PreAnalytiX) was extracted according to the manufacturer’s recommendations (PAXgene Blood RNA Kit, Qiagen) and the extracted total RNA was eluted in 40 μL of buffer BR5 and stored at − 80 °C.

For each sample, Cyanine-3 (Cy3) labeled cRNA was prepared from 200 ng of total RNA using the One-Color Quick Amp Labeling kit (Agilent) according to the manufacturer’s instructions, followed by RNeasy purification (QIAGEN). Dye incorporation and cRNA yield were checked with the Biospec nano spectrophotometer (Shimadzu). 600 ng of Cy3-labelled cRNA (specific activity> 6 pmol Cy3/μg cRNA) was fragmented at 60 °C for 30 minutes in a reaction volume of 25 μL containing 10x Agilent fragmentation buffer and 25x Agilent blocking agent following the manufacturer’s instructions. On completion of the fragmentation reaction, 25 μL of 2x Agilent hybridization buffer was added to the fragmentation mixture and hybridized to SurePrint G3 Mouse GE microarray (8X60K, Design 028005) enclosed in Agilent SureHyb-enabled hybridization chambers for 17 hours at 65 °C in a rotating Agilent hybridization oven. After hybridization, microarrays were washed sequentially in Wash buffer 1 (Agilent Technologies, 1 min), Wash buffer 2 (Agilent Technologies, 37 °C, 1 min). Slides were scanned immediately after washing on a Agilent G2505C Microarray Scanner with Agilent Scan Control A.8.5.1 software. The scanned images were analyzed with Feature Extraction Software 10.10.1.1 (Agilent) using default parameters (protocol GE1_1010_Sep10 and Grid: 037880_D_F_2012). All subsequent data analyses were done under R (www.r-project.org) using packages of Bioconductor (www.bioconductor.org). Raw data (median of the pixels intensity) was imported into R using the read.maimages function from the limma package [68]. Data were then stored in an ExpressionSet object and normalized by the quantile method using the normalize.quantiles function from the preprocessCore library.

### Transcriptome data

The porcine microarray (8 × 60 K, GPL16524, Agilent Technology) used in this experiment consisted of 43,603 probes derived from the 44 K Agilent-026440 porcine specific microarray (V2, for 71% of total probes), 3773 probes from the immune system, 9532 probes from adipose tissue and 3768 probes from muscle tissue, as already reported [69]. After quality control and quantile normalization steps [70], log2 transformation, data from 26 to 36 microarrays per tissue were available with 40,649 probes expressed in muscle, 37,276 probes in SCAT, 42,051 probes in liver, 39,072 probes in blood, 45,667 probes in pituitary, 46,097 probes in thyroid, 45,348 in adrenal glands for further statistical analysis. Normalized expression data (log2 transformed), raw data and sample information are available under NCBI GEO accession number GSE240257 as SuperSeries record including GSE240251 for total blood, GSE240253 for adrenal, GSE240254 for thyroid, GSE240255 for SCAT, GSE240345 for liver, GSE240347 for muscle and GSE240433 for pituitary. Annotation was based on Voillet et al. (2014) data [69] and was updated (nblast/Ensembl November 2018, Sscrofa11.1). Moreover, annotation for some differentially expressed probes (DEPs) was manually checked by a sequence alignment with blast against NCBI or Ensembl databases. 90.2% of DEPs were annotated.

### Data analysis

Data were analyzed using the R 4.2.1 software [66] with various packages from Bioconductor and CRAN.

### Blood dosage

A Kruskal-Wallis test was performed to test the hypothesis of a differential production between 32 °C and 24 °C for the blood dosages of the metabolites and hormones. The significance of the impact of HS on the three phenotypic variables (ST, RT and RR) was calculated with a Kruskal-Wallis and a Dunn test, as the normality hypothesis for ANOVA was not satisfied. A Student test was used for the comparison of the ADFI (by kg of body weight) between d-1 and d3 for the 18 pigs submitted to the challenge.

### Transcriptome data

A differential analysis was carried out separately for each dataset (7 transcriptomes) using the two packages *limma* [68] and the function dream from *variancePartition* [71, 72]. The package *limma* provides a standardized workflow for differential analysis with linear models on microarray data, while the addition of *dream* allows the use of linear mixed models, thus correcting for more than one effect. It is worth mentioning that *limma* can only take into account one random effect. Through testing, we choose to keep the following model (1), taking into account for the temperature (Temp), the breed, the climatic chamber (pen), the day of slaughter and the kinship within the breed.

1 $$y\:\sim\:0\:+\:Breed\:+\:Temp\:+\:\left(1\vert pen:\left(Temp:Breed\right)\right)\:+\:\left(1\vert slaughter:Temp\right)\:+\:\left(1\vert Father\right)\:+\:\left(1\vert Mother\right)$$

A Benjamini-Hochberg correction [73] was used and only genes with a corrected *p*-value (FDR) < 0.05 were considered as DEG. These were then used for performing a functional enrichment analysis with Gene Ontology (GO) [74], KEGG (Kyoto Encyclopedia of Genes and Genomes) [75] and Reactome [76] databases using the *gost* function from the package gprofiler2 [77]. Only the KEGG database was retained as it provides the most consistent results, and less redundancy. We choose to keep all the genes, up and down regulated for the analysis to have a better overview of the pathways involved. All the genes on the microarray were used as background for the analysis.

The enrichment networks were made using both the igraph package [78] and the visNetwork package [79] for visualization. Node color is the cluster detected with the *cluster fast greedy* function from the *igraph* package [78].

### Metabolome data

The same linear model as for the transcriptome was applied to the ASICS relative quantification of the 1H NMR data. As the packages used for the analysis of transcriptome data were not usable on our metabolome data, we used the model 1 with the lmer function of the R packages lme4 [80].

### Correlation between metabolic and transcriptomic data

A focus was made on the genes involved in the specific enrichment pathways of *oxidative phosphorylation, thermogenesis* and *chemical carcinogenesis-reactive oxygen species* in muscle and SCAT. For each DEG involved in these enrichments, a correlation was calculated with both the blood dosages and the differentially produced metabolites from the plasma, the urine and the associated tissue if available.

For the analysis, we kept only the genes and metabolites that displayed at least one absolute correlation> 0.5. For identified groups of genes, enrichment was made using GO and KEGG database with gprofiler2.

### Supplementary Information

**Additional file 1.** Table of the DEGs identified in the seven tissues. Cutoff for adjusted p-value was fixed at 0.05.

**Additional file 2.** Expression of the main genes expressed in the muscle discussed in the paper. For each gene, only the expression of the probe with the highest adjusted p-value is shown. PDHA1 = Pyruvate Dehydrogenase E1 Subunit Alpha 1, DLD = Dihydrolipoamide Dehydrogenase, PDK4 = Pyruvate Dehydrogenase Kinase 4, PYGM = Glycogen Phosphorylase, Muscle Associated, PHKA1 = Phosphorylase Kinase Regulatory Subunit Alpha 1, LDHB = Lactate Dehydrogenase B, BCAT1 = Branched Chain Amino Acid Transaminase 1, MLST8 = MTOR Associated Protein, LST8 Homolog, SOD1 = Superoxide Dismutase 1, PRDX4 = Peroxiredoxin 4, HIF1A = Hypoxia Inducible Factor 1 Subunit Alpha, GPX3 = Glutathione Peroxidase 3.

**Additional file 3.** Table of enrichments identified in the seven tissues. Pathways were enriched with GO, KEGG and Reactome databases. Cutoff for adjusted p-value was fixed at 0.05. A background corresponding to the annotated genes was used for multiple test p-value correction.

**Additional file 4.** Table of the differential analysis results for metabolomic data of four tissues (muscle, liver, plasma and urine). A total of 7, 8, 2, and 8 metabolites were differentially produced in muscle, liver, plasma, and urine, respectively (with an adjusted p-value of 0.05).

**Additional file 5: Figure S5.** Comparison of metabolites quantification by ASICS and blood dosage. The automatic quantification performs well on the metabolites both automatically identified and measured.

**Additional file 6: Figure S6.** Timeline of the different phenotypic measurements performed during the experiment. T°C Rec = Rectal temperature, HR = Hearth rate, T°C Cut = Skin temperature, RR = Respiratory rate.

**Additional file 7.** Table of kits for the measurement of blood metabolites and hormone levels.

## Data Availability

The metabolome datasets analysed during the current study are available in the Omics Dataverse repository from Recherche Data Gouv, with the DOI accession 10.57745/HCFXBY. The transcriptome datasets analysed during the current study are available in the NCBI GEO repository, with the accession number GSE240257.

## Abbreviations

TN: Thermoneutrality
HS: Heat Stress
ADFI: Average daily feed intake
DEG: Differentially Expressed Genes
SCAT: Subcutaneous Adipose Tissue
KEGG: Kyoto Encyclopedia of Genes and Genomes
GO: Gene Ontology
HSP: Heat Shock Proteins
LD: *Longissimus dorsi*
LDH: Lactate dehydrogenase
ROS: Reactive oxygen species
AA: Amino Acid

## References

[1] Cottrell JJ, Liu F, Hung AT, DiGiacomo K, Chauhan SS, Leury BJ (2015). Nutritional strategies to alleviate heat stress in pigs. Anim Prod Sci.

[2] St-Pierre NR, Cobanov B, Schnitkey G (2003). Economic losses from heat stress by US livestock Industries1. J Dairy Sci.

[3] da Fonseca de Oliveira AC, Vanelli K, Sotomaior CS, Weber SH, Costa LB (2019). Impacts on performance of growing-finishing pigs under heat stress conditions: a meta-analysis. Vet Res Commun.

[4] Renaudeau D, Gourdine JL, St-Pierre NR (2011). A meta-analysis of the effects of high ambient temperature on growth performance of growing-finishing pigs. J Anim Sci.

[5] Sanders SR, Cole LC, Flann KL, Baumgard LH, Rhoads RP. Effects of acute heat stress on skeletal muscle gene expression associated with energy metabolism in rats. FASEB J. 2009:598.7.

[6] Hao Y, Feng Y, Yang P, Cui Y, Liu J, Yang C (2016). Transcriptome analysis reveals that constant heat stress modifies the metabolism and structure of the porcine longissimus dorsi skeletal muscle. Mol Gen Genomics.

[7] Dou S, Villa-Vialaneix N, Liaubet L, Billon Y, Giorgi M, Gilbert H (2017). 1HNMR-based metabolomic profiling method to develop plasma biomarkers for sensitivity to chronic heat stress in growing pigs. PLoS One.

[8] Sanz Fernandez V, Johnson JS, Abuajamieh M, Stoakes SK, Seibert JT, Cox L (2015). Effects of heat stress on carbohydrate and lipid metabolism in growing pigs. Phys Rep.

[9] Collin A, Lebreton Y, Fillaut M, Vincent A, Thomas F, Herpin P (2001). Effects of exposure to high temperature and feeding level on regional blood flow and oxidative capacity of tissues in piglets. Exp Physiol.

[10] Gonzalez-Rivas PA, Chauhan SS, Ha M, Fegan N, Dunshea FR, Warner RD (2020). Effects of heat stress on animal physiology, metabolism, and meat quality: a review. Meat Sci.

[11] Horowitz M (2002). From molecular and cellular to integrative heat defense during exposure to chronic heat. Comp Biochem Physiol A Mol Integr Physiol.

[12] Sejian V, Bhatta R, Gaughan JB, Dunshea FR, Lacetera N (2018). Review: adaptation of animals to heat stress. Animal.

[13] Tardivel PJC, Canlet C, Lefort G, Tremblay-Franco M, Debrauwer L, Concordet D (2017). ASICS: an automatic method for identification and quantification of metabolites in complex 1D 1H NMR spectra. Metabolomics..

[14] Lefort G, Liaubet L, Canlet C, Tardivel P, Père M-C, Quesnel H (2019). ASICS: an R package for a whole analysis workflow of 1D 1H NMR spectra. Bioinformatics..

[15] Lefort G, Liaubet L, Marty-Gasset N, Canlet C, Vialaneix N, Servien R (2021). Joint automatic metabolite identification and quantification of a set of 1H NMR spectra. Anal Chem.

[16] Ingram DL (1965). Evaporative cooling in the pig. Nature..

[17] Huynh TTT, Aarnink AJA, Verstegen MWA, Gerrits WJJ, Heetkamp MJW, Kemp B (2005). Effects of increasing temperatures on physiological changes in pigs at different relative humidities1. J Anim Sci.

[18] Renaudeau D, Kerdoncuff M, Anaïs C, Gourdine JL (2008). Effect of temperature level on thermal acclimation in large white growing pigs. Animal..

[19] Collier RJ, Baumgard LH, Zimbelman RB, Xiao Y (2019). Heat stress: physiology of acclimation and adaptation. Animal Frontiers.

[20] Collier RJ, Gebremedhin KG (2015). Thermal biology of domestic animals. Annu Rev Anim Biosci.

[21] Milgen JV, Bernier JF, Lecozler Y, Dubois S, Noblet J (1998). Major determinants of fasting heat production and energetic cost of activity in growing pigs of different body weight and breed/castration combination*. Br J Nutr.

[22] Rhoads RP, Baumgard LH, Suagee JK (2013). Metabolic priorities during heat stress with an emphasis on skeletal muscle. J Anim Sci.

[23] Jang C, Hui S, Zeng X, Cowan AJ, Wang L, Chen L (2019). Metabolite exchange between mammalian organs quantified in pigs. Cell Metab.

[24] Collins FG, Mitros FA, Skibba JL (1980). Effect of palmitate on hepatic biosynthetic functions at hyperthermic temperatures. Metabolism..

[25] Lebret B, Serviento AM, Renaudeau D. Pork quality traits and associated muscle metabolic changes in pigs under chronic prenatal and postnatal heat stress. J Anim Sci. 2023:skad305. 10.1093/jas/skad305 PMC10629440 37708312

[26] Yang P, Hao Y, Feng J, Lin H, Feng Y, Wu X (2014). The expression of carnosine and its effect on the antioxidant capacity of longissimus dorsi muscle in finishing pigs exposed to constant heat stress. Asian Australas J Anim Sci.

[27] Goto T, Sugawara K, Nakamura S, Kidokoro S-I, Wakui H, Nunomura W (2016). Enzymatic and thermodynamic profiles of a heterotetramer lactate dehydrogenase isozyme in swine. Biochem Biophys Res Commun.

[28] Muller TL, Hewitt RJE, D’Souza DN, Barneveld RJ van, Muller TL, Hewitt RJE, et al. Factors influencing the measure of creatinine in non-reproductive pigs. Anim Prod Sci 2017;57:2418–2418.

[29] Ma B, Zhang L, Li J, Xing T, Jiang Y, Gao F (2021). Heat stress alters muscle protein and amino acid metabolism and accelerates liver gluconeogenesis for energy supply in broilers. Poult Sci.

[30] Ríus AG (2019). Invited review: adaptations of protein and amino acid metabolism to heat stress in dairy cows and other livestock species. Applied Animal Science.

[31] Bellego LL, van Milgen J, Noblet J (2002). Effect of high ambient temperature on protein and lipid deposition and energy utilization in growing pigs. Anim Sci.

[32] Kouba M, Hermier D, Le Dividich J (2001). Influence of a high ambient temperature on lipid metabolism in the growing pig. J Anim Sci.

[33] Serviento AM, Labussière E, Castex M, Renaudeau D (2020). Effect of heat stress and feeding management on growth performance and physiological responses of finishing pigs. J Anim Sci.

[34] Le Dividich J, Noblet J, Bikawa T (1987). Effect of environmental temperature and dietary energy concentration on the performance and carcass characteristics of growing-finishing pigs fed to equal rate of gain. Livest Prod Sci.

[35] Christon R (1988). The effect of tropical ambient temperature on growth and metabolism in Pigs1. J Anim Sci.

[36] Wu X, Li Z, Jia A, Su H, Hu C, Zhang M (2016). Effects of high ambient temperature on lipid metabolism in finishing pigs. J Integr Agric.

[37] Yousef MK, Johnson HD (1975). Thyroid activity in desert rodents: a mechanism for lowered metabolic rate. Am J Phys.

[38] Refetoff S, Feingold KR, Anawalt B, Blackman MR, Boyce A, Chrousos G, Corpas E (2000). Thyroid hormone serum transport proteins. Endotext.

[39] Li G, Ali IS, Currie RW (2006). Insulin induces myocardial protection and Hsp70 localization to plasma membranes in rat hearts. Am J Phys Heart Circ Phys.

[40] Pearce SC, Gabler NK, Ross JW, Escobar J, Patience JF, Rhoads RP (2013). The effects of heat stress and plane of nutrition on metabolism in growing pigs1. J Anim Sci.

[41] Sanz Fernandez MV, Stoakes SK, Abuajamieh M, Seibert JT, Johnson JS, Horst EA (2015). Heat stress increases insulin sensitivity in pigs. Phys Rep.

[42] O’Brien MD, Rhoads RP, Sanders SR, Duff GC, Baumgard LH (2010). Metabolic adaptations to heat stress in growing cattle. Domest Anim Endocrinol.

[43] Maloyan A, Palmon A, Horowitz M (1999). Heat acclimation increases the basal HSP72 level and alters its production dynamics during heat stress. Am J Phys Regul Integr Comp Phys.

[44] Bambou J-C, Gourdine J-L, Grondin R, Vachiery N, Renaudeau D (2011). Effect of heat challenge on peripheral blood mononuclear cell viability: comparison of a tropical and temperate pig breed. Trop Anim Health Prod.

[45] Pennarossa G, Maffei S, Rahman MM, Berruti G, Brevini TAL, Gandolfi F (2012). Characterization of the constitutive pig ovary heat shock chaperone machinery and its response to acute thermal stress or to seasonal Variations1. Biol Reprod.

[46] Dangi SS, Gupta M, Dangi SK, Chouhan VS, Maurya VP, Kumar P (2015). Expression of HSPs: an adaptive mechanism during long-term heat stress in goats (Capra hircus). Int J Biometeorol.

[47] Li L, Wu J, Luo M, Sun Y, Wang G (2016). The effect of heat stress on gene expression, synthesis of steroids, and apoptosis in bovine granulosa cells. Cell Stress Chaperones.

[48] Cervantes M, Cota M, Arce N, Castillo G, Avelar E, Espinoza S (2016). Effect of heat stress on performance and expression of selected amino acid and glucose transporters, HSP90, leptin and ghrelin in growing pigs. J Therm Biol.

[49] Poullet N, Devarieux O, Beramice D, Dantec L, Félicité Y, Feuillet D (2023). Comparative analysis of whole blood transcriptomics between European and local Caribbean pigs in response to feed restriction in a tropical climate. BMC Genomics.

[50] Azad MAK, Kikusato M, Sudo S, Amo T, Toyomizu M (2010). Time course of ROS production in skeletal muscle mitochondria from chronic heat-exposed broiler chicken. Comp Biochem Physiol A Mol Integr Physiol.

[51] Montilla SIR, Johnson TP, Pearce SC, Gardan-Salmon D, Gabler NK, Ross JW (2014). Heat stress causes oxidative stress but not inflammatory signaling in porcine skeletal muscle. Temperature.

[52] Stowe DF, Camara AKS (2009). Mitochondrial reactive oxygen species production in excitable cells: modulators of mitochondrial and cell function. Antioxid Redox Signal.

[53] Xia B, Wu W, Fang W, Wen X, Xie J, Zhang H (2022). Heat stress-induced mucosal barrier dysfunction is potentially associated with gut microbiota dysbiosis in pigs. Animal Nutrition.

[54] Cui Y, Wang C, Hao Y, Gu X, Wang H (2019). Chronic heat stress induces acute phase responses and serum metabolome changes in finishing pigs. Animals.

[55] Cruzen SM, Pearce SC, Baumgard LH, Gabler NK, Huff-Lonergan E, Lonergan SM (2015). Proteomic changes to the sarcoplasmic fraction of predominantly red or white muscle following acute heat stress. J Proteome.

[56] Stremler KE, Stafforini DM, Prescott SM, McIntyre TM (1991). Human plasma platelet-activating factor acetylhydrolase. Oxidatively fragmented phospholipids as substrates. J Biol Chem.

[57] Lackner J, Albrecht A, Mittler M, Marx A, Kreyenschmidt J, Hess V (2021). Effect of feeding histidine and β-alanine on carnosine concentration, growth performance, and meat quality of broiler chickens. Poult Sci.

[58] Pearce SC, Lonergan SM, Huff-Lonergan E, Baumgard LH, Gabler NK (2015). Acute heat stress and reduced nutrient intake Alter intestinal proteomic profile and gene expression in pigs. PLoS One.

[59] Cheng C-Y, Tu W-L, Chen C-J, Chan H-L, Chen C-F, Chen H-H (2018). Functional genomics study of acute heat stress response in the small yellow follicles of layer-type chickens. Sci Rep.

[60] Blincoe C, Brody S, Burge G, Tuner HG, Worstell D, Elliot JR. Environmental physiology with special reference to domestic animals. 17. The influence of temperature on blood composition of cattle. Environmental physiology with special reference to domestic animals 17 The influence of temperature on blood composition of cattle. 1951.

[61] Tanaka M, Kamiya Y, Kamiya M, Nakai Y (2007). Effect of high environmental temperatures on ascorbic acid, sulfhydryl residue and oxidized lipid concentrations in plasma of dairy cows. Anim Sci J.

[62] Rejeb M, Sadraoui R, Najar T (2016). Role of vitamin C on immune function under heat stress condition in dairy cows. Asian Journal of Animal and Veterinary Advances.

[63] Sahin K, Sahin N, Kucuk O (2003). Effects of chromium, and ascorbic acid supplementation on growth, carcass traits, serum metabolites, and antioxidant status of broiler chickens reared at a high ambient temperature (32°C). Nutr Res.

[64] Padilla L, Matsui T, Kamiya Y, Kamiya M, Tanaka M, Yano H (2006). Heat stress decreases plasma vitamin C concentration in lactating cows. Livest Sci.

[65] Renaudeau D, Anais C, Tel L, Gourdine JL (2010). Effect of temperature on thermal acclimation in growing pigs estimated using a nonlinear function. J Anim Sci.

[66] R: A Language and Environment for Statistical Computing. 2022.

[67] Martin M, Legat B, Leenders J, Vanwinsberghe J, Rousseau R, Boulanger B (2018). PepsNMR for 1H NMR metabolomic data pre-processing. Anal Chim Acta.

[68] Ritchie ME, Phipson B, Wu D, Hu Y, Law CW, Shi W (2015). Limma powers differential expression analyses for RNA-sequencing and microarray studies. Nucleic Acids Res.

[69] Voillet V, SanCristobal M, Lippi Y, Martin PG, Iannuccelli N, Lascor C (2014). Muscle transcriptomic investigation of late fetal development identifies candidate genes for piglet maturity. BMC Genomics.

[70] Bolstad BM, Irizarry RA, Astrand M, Speed TP (2003). A comparison of normalization methods for high density oligonucleotide array data based on variance and bias. Bioinformatics.

[71] Hoffman GE, Schadt EE (2016). variancePartition: interpreting drivers of variation in complex gene expression studies. BMC Bioinformatics.

[72] Hoffman GE, Roussos P (2021). Dream: powerful differential expression analysis for repeated measures designs. Bioinformatics.

[73] Benjamini Y, Hochberg Y (1995). Controlling the false discovery rate: a practical and powerful approach to multiple testing. J R Stat Soc Ser B Methodol.

[74] Ashburner M, Ball CA, Blake JA, Botstein D, Butler H, Cherry JM (2000). Gene ontology: tool for the unification of biology. Nat Genet.

[75] Kanehisa M, Goto S (2000). KEGG: Kyoto encyclopedia of genes and genomes. Nucleic Acids Res.

[76] Gillespie M, Jassal B, Stephan R, Milacic M, Rothfels K, Senff-Ribeiro A (2022). The reactome pathway knowledgebase 2022. Nucleic Acids Res.

[77] Kolberg L, Raudvere U, Kuzmin I, Vilo J, Peterson H. gprofiler2 -- an R package for gene list functional enrichment analysis and namespace conversion toolset g:Profiler. 2020. 10.12688/f1000research.24956.1 PMC7859841 33564394

[78] Csardi G, Nepusz T. The igraph software package for complex network research. InterJournal. 2006;Complex Systems:1695.

[79] htmlwidgets/lib ABV and C (vis js library in, https://visjs.org, https://github.com/visjs/vis-network), interface) BT (R. visNetwork: Network Visualization using “vis.js” Library. 2022.

[80] Bates D, Mächler M, Bolker B, Walker S (2015). Fitting linear mixed-effects models using lme4. J Stat Softw.

[81] Sert NP du, Hurst V, Ahluwalia A, Alam S, Avey MT, Baker M, et al. The ARRIVE guidelines 2.0: updated guidelines for reporting animal research. PLoS Biol 2020;18:e3000410. 10.1371/journal.pbio.3000410 PMC7360023 32663219

