# Supplementary figures and images for: Multi-tissue metabolic and transcriptomic responses to a short-term heat stress in swine
Source: BMC Genomics. 2024 Jan 23;25:99. doi: 10.1186/s12864-024-09999-1 (PMC10804606; doi:10.1186/s12864-024-09999-1)

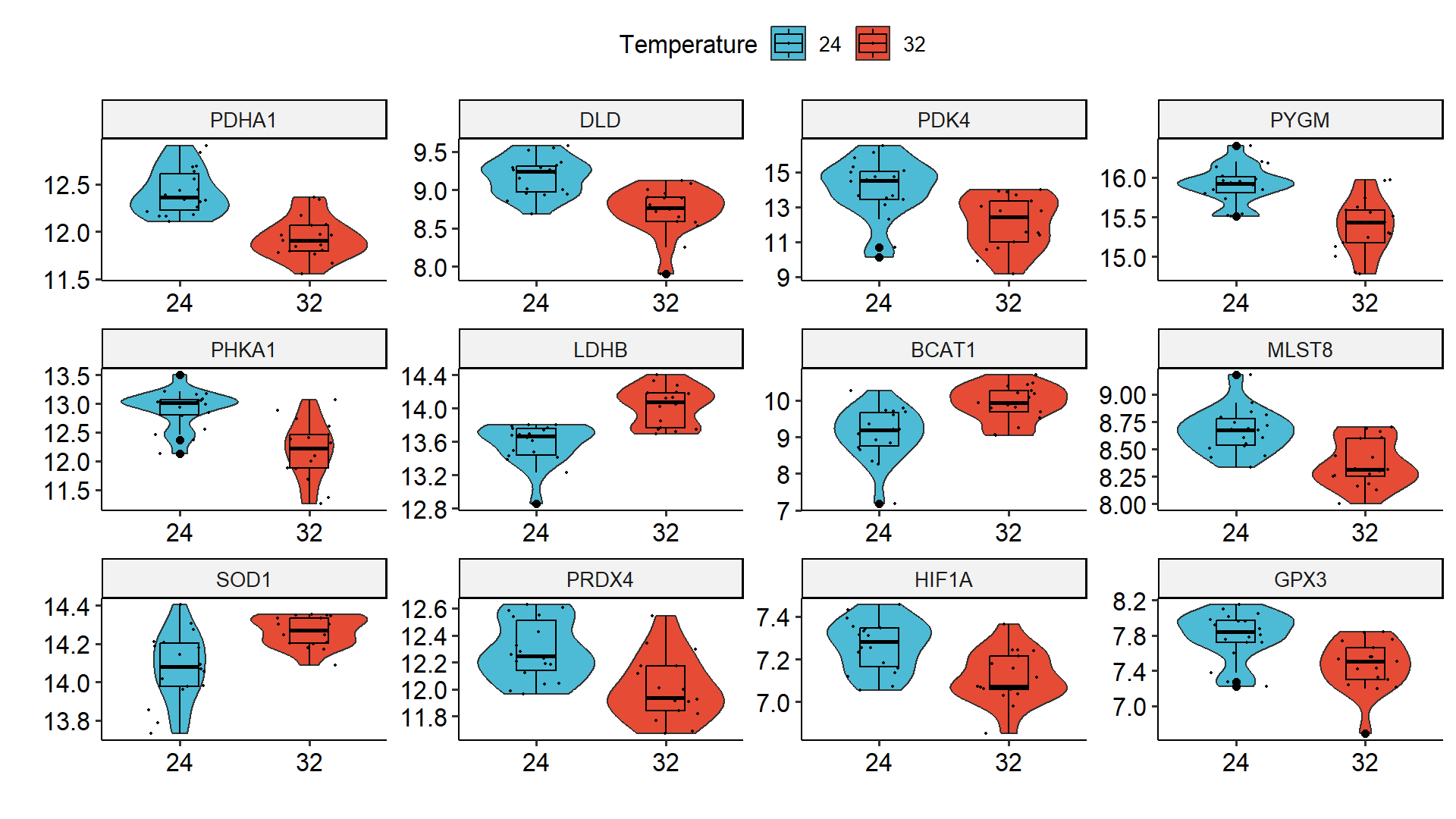

Supplement: Supplementary file 2 — Additional file 2. Expression of the main genes expressed in the muscle discussed in the paper. For each gene, only the expression of the probe with the highest adjusted p-value is shown. PDHA1 = Pyruvate Dehydrogenase E1 Subunit Alpha 1, DLD = Dihydrolipoamide Dehydrogenase, PDK4 = Pyruvate Dehydrogenase Kinase 4, PYGM = Glycogen Phosphorylase, Muscle Associated, PHKA1 = Phosphorylase Kinase Regulatory Subunit Alpha 1, LDHB = Lactate Dehydrogenase B, BCAT1 = Branched Chain Amino Acid Transaminase 1, MLST8 = MTOR Associated Protein, LST8 Homolog, SOD1 = Superoxide Dismutase 1, PRDX4 = Peroxiredoxin 4, HIF1A = Hypoxia Inducible Factor 1 Subunit Alpha, GPX3 = Glutathione Peroxidase 3. [file 12864_2024_9999_MOESM2_ESM.png]

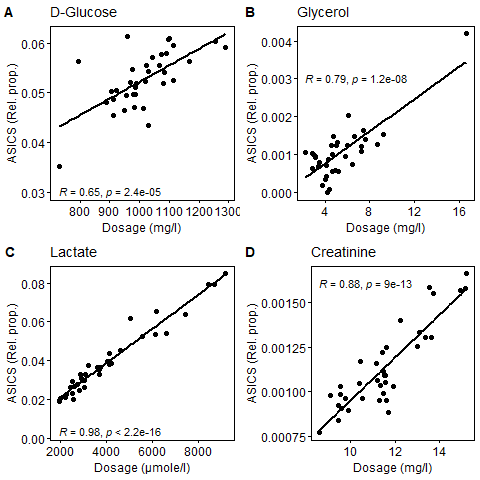

Supplement: Supplementary file 5 — Additional file 5: Figure S5. Comparison of metabolites quantification by ASICS and blood dosage. The automatic quantification performs well on the metabolites both automatically identified and measured. [file 12864_2024_9999_MOESM5_ESM.png]

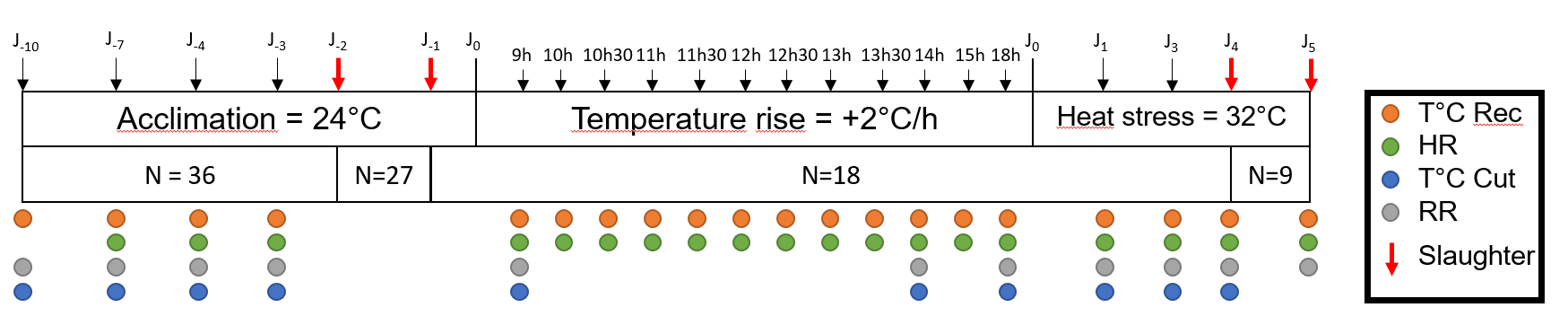

Supplement: Supplementary file 6 — Additional file 6: Figure S6. Timeline of the different phenotypic measurements performed during the experiment. T°C Rec = Rectal temperature, HR = Hearth rate, T°C Cut = Skin temperature, RR = Respiratory rate. [file 12864_2024_9999_MOESM6_ESM.png]
